# A Dysbiosis–Urolithin A Depletion–Low 6-Sulfatoxymelatonin Triad as a Composite Biomarker Framework Across Age-Related Vascular and Malignant Disease States

**DOI:** 10.3390/ijms27135753

**Published:** 2026-06-25

**Authors:** Alexandre Tavartkiladze, Levan Tavartkiladze, Gaiane Simonia, Michel Burnier, Russel J. Reiter, Ruite Lou, Dinara Kasradze, Nana Okrostsvaridze, Pati Revazishvili, Irine Andronikashvili, Pirdara Nozadze, Rusudan Khutsishvili

**Affiliations:** 1 Department of Internal Medicine, Faculty of Medicine, Tbilisi State Medical University, 0186 Tbilisi, Georgia; gsimonia@gmail.com (G.S.); pati_revazishvili@yahoo.com (P.R.); i.andronikashvili@tsmu.edu (I.A.); p.nozadze@gmail.com (P.N.); 2 Institute for Personalized Medicine, 0186 Tbilisi, Georgia; levan001@gmail.com (L.T.); dinarakasradze@yahoo.com (D.K.); nana_oqro@yahoo.com (N.O.); feria.xucishvili@gmail.com (R.K.); 3 Department of Biotechnology, Foconsci Chemical Industry, Shaoxing 312000, China; sandrotavphd@yahoo.fr; 4 Service of Nephrology and Hypertension, Department of Medicine, Lausanne University Hospital, 1011 Lausanne, Switzerland; michel.burnier@netplus.ch; 5 Department of Cellular & Structural Biology, University of Texas Health Science Center, San Antonio, TX 78229, USA; reiter@uthscsa.edu

**Keywords:** urolithin A, gut microbiota, dysbiosis, salt-sensitive hypertension, melatonin, 6-sulfatoxymelatonin, cytokines, oxidative stress, inflammaging, advanced cancer, composite biomarker triad

## Abstract

Urolithin A (UA) is a gut microbiota-derived postbiotic generated from dietary ellagitannins, while urinary 6-sulfatoxymelatonin (6-SMT) is a surrogate marker of nocturnal melatonin output. We explored whether dysbiosis, UA depletion, and low 6-SMT define an ordered biomarker pattern across healthy aging, salt-sensitive hypertension, and advanced cancer. In this pilot observational study, patients with advanced solid tumors were classified as Cancer Group 1 (higher aggressiveness; *n* = 231) or Cancer Group 2 (lower aggressiveness; *n* = 118) and compared with healthy older controls (*n* = 117) and elderly participants with salt-sensitive hypertension (*n* = 333). Plasma UA decreased stepwise from controls (2.24 [1.38–3.12] nmol/L) to salt-sensitive hypertension (1.20 [0.76–1.89] nmol/L), Cancer Group 2 (0.60 [0.32–0.91] nmol/L), and Cancer Group 1, in which most samples were at or below the assay limit (overall *p* < 0.0001; trend *p* < 0.0001). Twenty-four-hour urinary 6-SMT declined in parallel, whereas the dysbiosis score, interleukin-6 (IL-6), tumor necrosis factor-alpha (TNF-α), interleukin-8 (IL-8), and malondialdehyde (MDA) increased, and antioxidant indices decreased across the ordered groups (all *p* < 0.0001). These cross-sectional findings support a hypothesis-generating biomarker framework linking dysbiosis, postbiotic depletion, circadian disruption, inflammation, and redox imbalance across age-related disease states.

## 1. Introduction

The human gut microbiota mediates the biotransformation of dietary polyphenols into bioactive metabolites, shaping host metabolic and immune phenotypes. Ellagitannin- and ellagic acid-rich foods (e.g., pomegranate, walnuts, berries) are metabolized to urolithins, among which urolithin A (UA) is often the dominant circulating metabolite in urolithin-producing individuals [1,2,3].

UA has been reported to modulate inflammatory signaling and mitochondrial quality control pathways in experimental systems, but its systemic availability is highly dependent on inter-individual variation in microbiota composition and function (‘urolithin metabotypes’) [1,2,3,4,5,6,7,8,9]. Loss of urolithin-producing capacity may therefore serve as a functional readout of microbiome collapse relevant to aging-associated disorders.

Advanced malignancy is increasingly recognized as a systemic disorder that extends beyond tumor genotype to include host metabolism, inflammation, and microbiome remodeling. Cancer-associated dysbiosis can alter microbial metabolite production and may suppress the conversion of ellagic acid/ellagitannins to UA [10,11,12].

Among the taxa implicated in gut ecosystem stability and polyphenol biotransformation, *Streptococcus thermophilus* and *Enterococcus faecium* merit particular attention. *S. thermophilus* contributes to exopolysaccharide production and short-chain fatty acid metabolism [13,14], while *E. faecium*, despite its dual identity as a commensal and opportunistic pathogen, has documented probiotic properties relevant to mucosal immune modulation [15].

Circadian disruption and impaired melatonin signaling are common in advanced disease and may contribute to immune dysregulation, sleep disturbance, and metabolic stress. Twenty-four-hour urinary 6-sulfatoxymelatonin (6-SMT), the main urinary metabolite of melatonin, is widely used as a surrogate of nocturnal melatonin output [16,17,18,19,20,21,22].

Salt-sensitive hypertension represents a clinically relevant age-related comparator characterized by vascular stress, oxidative imbalance, low-grade inflammation, and altered sodium handling. In the present study, it is not considered a premalignant state but a biologically informative intermediate context in which a microbiome-linked metabolic and circadian signature may also be detectable. This rationale is used here as a hypothesis-generating framework rather than as proof of a linear mechanistic continuum.

We hypothesized that (i) advanced cancer with higher clinical aggressiveness would show the greatest depletion of systemic and fecal UA; (ii) this depletion would co-occur with selective dysbiosis and reduced 6-SMT; (iii) inflammatory and redox markers would shift in parallel with these changes; and (iv) salt-sensitive hypertension would show an intermediate, hypothesis-generating pattern between healthy older controls and advanced cancer. Because the design is observational, these hypotheses were examined as ordered associations rather than causal relationships.

## 2. Results

### 2.1. Cohort Characteristics and Specimen Availability

**Figure 1:**
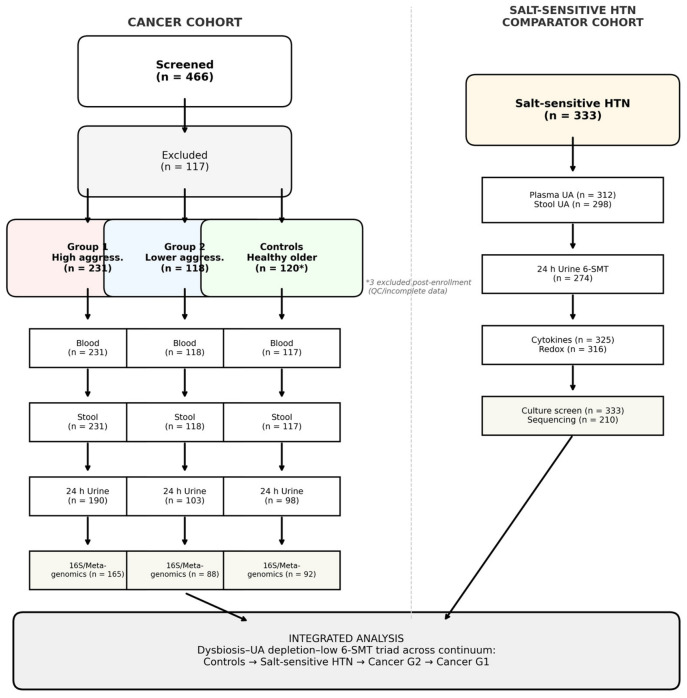
Study design and specimen availability across cohorts. Left: cancer cohort (*n* = 466 screened, 349 enrolled; stratified into Cancer Group 1 [higher aggressiveness, *n* = 231], Cancer Group 2 [lower aggressiveness, *n* = 118], and healthy older controls [*n* = 120 enrolled, *n* = 117 after QC exclusion]). Right: salt-sensitive hypertension comparator cohort (*n* = 333). Assay-specific specimen availability is shown for each biospecimen type, including the sequencing subset. Abbreviations: UA, urolithin A; 6-SMT, 6-sulfatoxymelatonin; HTN, hypertension; QC, quality control.

A total of 349 patients with advanced solid tumors and 117 healthy older controls were included in the cancer-cohort analysis. Oncology participants were stratified into Cancer Group 1 (higher aggressiveness; *n* = 231) and Cancer Group 2 (lower aggressiveness; *n* = 118). In the comparator cohort, 333 elderly individuals with salt-sensitive hypertension were analyzed. Figure 1 summarizes participant flow and assay-specific specimen availability.

### 2.2. Urolithin A Is Profoundly Depleted in Aggressive Advanced Cancer and Intermediate in Salt-Sensitive Hypertension

Plasma UA (nmol/L) differed significantly across the four groups (overall *p* < 0.0001; trend *p* < 0.0001). Healthy older controls showed the highest concentrations; salt-sensitive hypertension showed an intermediate reduction; Cancer Group 2 showed further depletion; and Cancer Group 1 showed values at or below the assay limit in most samples. Stool UA (nmol/g) showed a concordant stepwise pattern. Full quantitative distributions are provided in Table 1.

**Figure 2:**
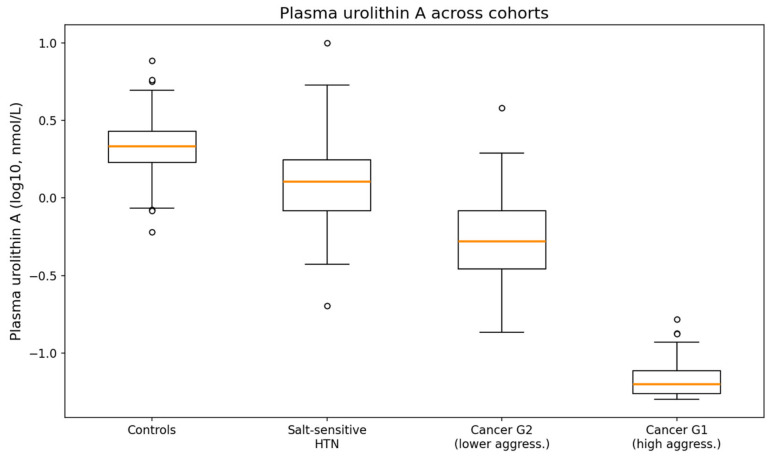
Plasma urolithin A (UA) across cohorts (healthy older controls, salt-sensitive hypertension, Cancer Group 2, and Cancer Group 1). In each boxplot, the orange horizontal line denotes the median, the box denotes the interquartile range (IQR), the whiskers indicate non-outlier values, and circles denote outliers; overall *p* < 0.0001 and trend *p* < 0.0001; assay-specific *n* values are reported in Table 1.

As shown in Figure 2, plasma urolithin A decreases stepwise across the ordered disease-state gradient (healthy older controls → salt-sensitive hypertension → Cancer Group 2 → Cancer Group 1), supporting a graded loss of microbiota-derived postbiotic capacity.

### 2.3. Reduced 24 h Urinary 6-SMT Tracks with Disease Severity

Twenty-four-hour urinary 6-SMT (ng/day) declined stepwise across healthy older controls, salt-sensitive hypertension, Cancer Group 2, and Cancer Group 1 (overall *p* < 0.0001; trend *p* < 0.0001; Table 1). The most pronounced reduction was observed in Cancer Group 1, whereas salt-sensitive hypertension retained an intermediate pattern.

**Figure 3:**
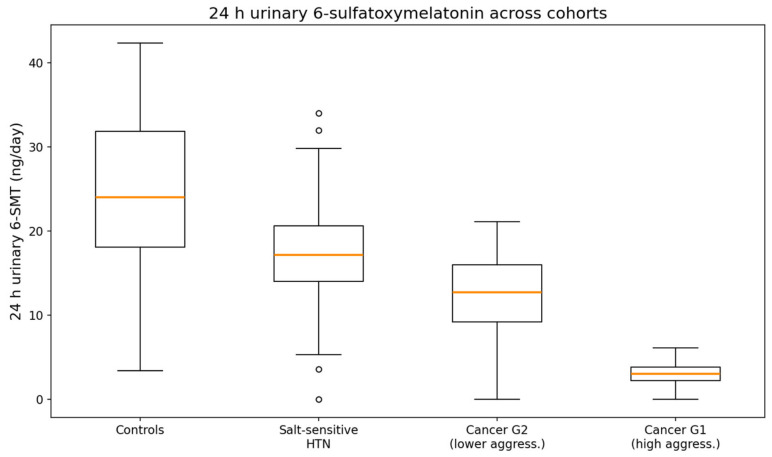
24 h urinary 6-sulfatoxymelatonin (6-SMT; ng/day) across cohorts (healthy older controls, salt-sensitive hypertension, Cancer Group 2, and Cancer Group 1). In each boxplot, the orange horizontal line denotes the median, the box denotes the IQR, the whiskers indicate non-outlier values, and circles denote outliers; overall *p* < 0.0001 and trend *p* < 0.0001; assay-specific *n* values are reported in Table 1.

This progressive decline in 24 h urinary 6-SMT is visualized in Figure 3 and mirrors the ordered disease-state gradient.

### 2.4. Dysbiosis Patterns and Inflammatory Cytokines

Culture-based screening and sequencing-based profiling supported a stepwise increase in dysbiosis across the ordered groups. The composite dysbiosis score increased from healthy older controls to salt-sensitive hypertension and then to Cancer Group 2 and Cancer Group 1. Phylum-level shifts were concordant, with decreasing Bacteroidetes and increasing Proteobacteria. Interleukin-6 (IL-6), tumor necrosis factor-alpha (TNF-α), and interleukin-8 (IL-8) increased in parallel, whereas interleukin-10 (IL-10) remained comparatively preserved. Full quantitative distributions are provided in Table 1.

### 2.5. Redox Imbalance Supports Mitochondrial–Lysosomal Stress in Intermediate and Advanced Disease

Oxidative stress and antioxidant indices also changed stepwise across the ordered groups. Malondialdehyde (MDA) increased progressively, whereas total antioxidant capacity (TAC), reduced-to-oxidized glutathione ratio (GSH/GSSG), superoxide dismutase (SOD), and catalase decreased, with the most pronounced imbalance in Cancer Group 1 (Table 1).

**Figure 4:**
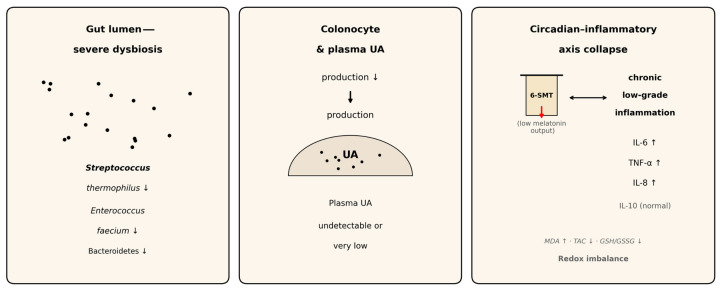
Proposed mechanistic model linking gut dysbiosis to circadian–redox–immune axis collapse: (**Left panel**) Selective depletion of ecosystem-stabilizing taxa (*Streptococcus thermophilus*, *Enterococcus faecium*, and Bacteroidetes). (**Middle panel**) Loss of colonocyte urolithin A production leading to undetectable plasma UA. (**Right panel**) Convergent circadian disruption (low 6-SMT), chronic low-grade inflammation (IL-6/TNF-α/IL-8 elevated with preserved IL-10), and progressive redox imbalance (MDA ↑, TAC ↓, GSH/GSSG ↓). Vertical arrows indicate the direction of the proposed sequence or reduction; ↑ and ↓ denote increases and decreases, respectively; ↔ denotes a bidirectional association.

A synthesized mechanistic interpretation of the dysbiosis–UA depletion–low 6-SMT axis and its links to inflammatory and redox amplification is presented in Figure 4.

### 2.6. Proposal of a Composite Dysbiosis–UA Depletion–Low 6-SMT Triad Framework

Integrating dysbiosis indicators, plasma/stool UA, and urinary 6-SMT, we propose a composite biomarker triad that captures a microbiome-linked metabolic–circadian disruption state. These categories are intended as a hypothesis-generating interpretive framework rather than a validated clinical classification.

The operational triad categories are summarized in Table 2. In the present dataset, the most disrupted pattern was observed predominantly in Cancer Group 1, intermediate patterns in salt-sensitive hypertension and Cancer Group 2, and the least disrupted pattern in healthy older controls.

## 3. Discussion

This study proposes an integrated microbiome–postbiotic–circadian–redox framework across age-related disease states, using salt-sensitive hypertension as a comparator and advanced cancer as the most amplified clinical context in the present dataset. Higher-aggressiveness advanced cancer was associated with profound depletion of systemic and fecal UA, more marked dysbiosis, lower 6-SMT, higher pro-inflammatory cytokines, and worsening redox imbalance.

The choice of salt-sensitive hypertension was conceptual and hypothesis-generating. This condition shares vascular stress, oxidative imbalance, low-grade inflammation, and altered sodium handling with other age-related disorders, yet it is distinct from cancer and should not be interpreted as a premalignant stage. In our data, salt-sensitive hypertension consistently occupied an intermediate position across multiple biomarkers, which made it a useful comparator for exploring an ordered host–microbe–metabolite gradient.

Mechanistically, UA depletion may represent loss of microbiome-dependent polyphenol biotransformation, whereas reduced 6-SMT may reflect circadian and endocrine disruption. These observations are compatible with a model in which dysbiosis, postbiotic depletion, inflammation, and oxidative stress cluster together, but the present design does not allow causal inference.

From a translational perspective, the proposed triad should be viewed as a research framework rather than a routine clinical panel. Plasma and stool UA measurement, 24 h urinary 6-SMT assessment, and microbiome profiling require specialized workflows and cross-platform standardization. Their near-term utility is therefore most plausible in research stratification, mechanistic studies, and prospective validation.

Key limitations include the cross-sectional observational design; incomplete harmonization of potential confounders such as diet, sleep, antibiotic exposure, treatment effects, and medication use; and the descriptive nature of the present statistical analysis. In particular, antihypertensive medication exposure, including beta-blockers, may influence melatonin-related readouts and is therefore acknowledged as a potential confounder. The cancer aggressiveness grouping is also exploratory and should not be interpreted as a validated prognostic score.

Future work should include longitudinal sampling, dataset-verified confounder adjustment, and functional studies testing whether restoration of urolithin-producing capacity or melatonin signaling tracks with improvement in inflammatory and redox readouts. Prospective validation will be required before any mechanistic or prognostic use of the proposed framework can be considered.

## 4. Materials and Methods

### 4.1. Study Design and Cohorts

Two observational source cohorts were analyzed, and four analytic groups were compared in the present manuscript: healthy older controls (*n* = 117), salt-sensitive hypertension (*n* = 333), Cancer Group 2 (lower aggressiveness; *n* = 118), and Cancer Group 1 (higher aggressiveness; *n* = 231).

(A) Advanced solid tumor cohort: A three-year pilot observational study (January 2018–December 2020) was conducted at the GA Cancer Center (Tbilisi, Georgia) and the Institute for Personalized Medicine (Tbilisi, Georgia). Adults aged 57–85 years with confirmed stage IV solid tumors were eligible if they could provide blood and stool samples; 24 h urine was collected where feasible.

(B) Salt-sensitive hypertension comparator cohort: Elderly adults aged 57–85 years with salt-sensitive hypertension were evaluated using the same biomarker axis. Healthy older controls were adults of similar age without known malignancy or arterial hypertension.

Healthy older controls were required to have no current antihypertensive treatment, no recent systemic antibiotic exposure, and no active inflammatory condition expected to materially alter microbiome or melatonin-related measures. The study is reported as observational and hypothesis-generating; a completed Strengthening the Reporting of Observational Studies in Epidemiology (STROBE) checklist is provided in the Supplementary Materials Tables S1–S3.

### 4.2. Clinical Definitions and Stratification

Oncology participants were stratified descriptively as Cancer Group 1 (higher aggressiveness) or Cancer Group 2 (lower aggressiveness) based on tumor type and overall clinical course. This grouping was used to organize the exploratory biomarker analysis and is not presented as a validated prognostic classification (Table 3).

Salt sensitivity of blood pressure was defined using a standardized protocol with low-salt and high-salt exposure and repeated blood pressure measurements; salt sensitivity was considered present when the difference in mean arterial pressure exceeded 3 mmHg, with repeated 24 h urinary sodium excretion used to assess dietary compliance [23,24,25].

### 4.3. Biospecimen Collection, Processing, and Storage

Blood was collected in the morning (08:00–10:00) under standardized fasting conditions (minimum 8 h overnight fast). Plasma (for UA) and serum (for cytokines and redox markers) were processed within 60 min and stored at −80 °C until analysis.

Stool was split into aliquots for targeted urolithin analysis and microbiome assessment (culture-based screening and sequencing) and stored at −80 °C.

For urinary 6-SMT, 24 h urine collections were obtained using a standardized start/stop protocol; total volume was recorded, mixed, aliquoted, and stored at −80 °C.

### 4.4. Targeted Quantification of Urolithin A in Plasma and Stool

UA was quantified by high-performance liquid chromatography (HPLC) using an authentic reference standard, with plasma analyzed as total UA after enzymatic hydrolysis to capture conjugated forms. Calibration, limits of detection/quantification, and quality assurance/quality control (QA/QC) procedures are summarized in Supplementary Methods and were aligned with minimum reporting expectations for chemical analysis [26,27].

### 4.5. Urinary 6-Sulfatoxymelatonin

Urinary 6-SMT was quantified from 24 h urine collections using a validated immunoassay. Results are expressed as total daily excretion (ng/day) by multiplying the measured concentration by the 24 h urine volume. Creatinine was used to support plausibility checks for collection completeness.

### 4.6. Serum Cytokines

Serum IL-6, TNF-α, IL-8, and IL-10 were measured using commercially available immunoassays according to manufacturers’ instructions. Samples were assayed in duplicate, and plate acceptance criteria were applied using internal quality controls.

### 4.7. Oxidative and Antioxidant Status

Oxidative stress and antioxidant defense were assessed using a panel comprising malondialdehyde (MDA), total antioxidant capacity (TAC), reduced/oxidized glutathione ratio (GSH/GSSG), superoxide dismutase (SOD), and catalase activity. Assays were performed on serum or plasma using validated colorimetric/fluorometric methods; all reagents were sourced from Sigma-Aldrich (Burlington, MA, USA) (kit and instrument details provided in Supplementary Methods).

### 4.8. Microbiome Assessment

Dysbiosis was evaluated using two complementary approaches:

(i) Culture-based targeted screening for key taxa and ecosystem indicators (including *Streptococcus thermophilus*, *Enterococcus faecium*, and representatives of Bacteroidetes and Proteobacteria).

(ii) Sequencing-based profiling (16S ribosomal RNA gene sequencing or metagenomics, depending on sample availability) to estimate alpha/beta diversity and relative abundances. Bioinformatic pipelines, reference databases, and QC thresholds are provided in Supplementary Methods.

Culture-based results were interpreted as indicators of pronounced selective dysbiosis rather than comprehensive community profiling.

Analyses were primarily descriptive and hypothesis-generating. Continuous variables are reported as median [interquartile range (IQR)] or mean [standard deviation (SD)], as appropriate. Between-group comparisons used nonparametric tests (Kruskal–Wallis with post hoc Dunn tests) or χ^2^/Fisher’s exact tests for categorical variables.

To evaluate the ordered pattern across disease states, trend tests and correlation analyses among UA, 6-SMT, dysbiosis indices, cytokines, and redox markers were performed. Because clinically relevant covariates such as detailed diet, sleep measures, treatment exposures, and medication-class data were not fully harmonized across both source cohorts in the current revision files, adjusted multivariable models are not emphasized here, and the results are interpreted as exploratory.

Two-sided *p*-values < 0.05 were considered statistically significant.

## 5. Conclusions

Across healthy older controls, salt-sensitive hypertension, and advanced solid tumors, we observed a graded association linking dysbiosis, UA depletion, reduced 6-SMT, inflammatory activation, and redox imbalance. These cross-sectional findings support a hypothesis-generating biomarker framework but require prospective and mechanistic validation before causal or clinical conclusions can be drawn.

## Figures and Tables

**Table 1:** Quantitative biomarker distributions across cohorts. Values are median [interquartile range (IQR)] for continuous variables and % (*n*/N) for categorical variables; *p*-values are from Kruskal–Wallis (continuous) or χ^2^ (categorical), with a trend *p*-value across the ordered groups (healthy older controls → salt-sensitive hypertension → Cancer Group 2 → Cancer Group 1). Assay-specific *n* values are reported per biomarker due to differential biospecimen availability and QC exclusions.

Biomarker (Unit)	Controls (*n*)	Salt-Sensitive HTN (*n*)	Cancer Group 2 (*n*)	Cancer Group 1 (*n*)	*p* (Overall)	*p* (Trend)
Plasma UA (nmol/L)	2.24 [1.38–3.12] (117)	1.20 [0.76–1.89] (312)	0.60 [0.32–0.91] (118)	0.05 [0.05–0.05] (231)	<0.0001	<0.0001
Stool UA (nmol/g)	16.87 [10.14–25.12] (117)	9.76 [5.59–15.77] (298)	5.01 [3.47–7.72] (118)	0.50 [0.50–0.50] (231)	<0.0001	<0.0001
24 h urinary 6-SMT (ng/day)	24.39 [18.89–30.44] (98)	17.70 [14.48–21.74] (274)	12.17 [9.07–15.92] (103)	3.04 [2.55–3.94] (190)	<0.0001	<0.0001
Dysbiosis score (0–10)	1.52 [1.08–2.13] (117)	3.68 [2.78–4.73] (333)	4.94 [4.07–6.25] (118)	7.34 [6.20–8.46] (231)	<0.0001	<0.0001
Urolithin- producing taxa detected (%)	77.8% (91/117)	51.6% (160/310)	23.7% (28/118)	9.1% (21/231)	<0.0001	<0.0001
Bacteroidetes (%)	30.07 [25.27–35.58] (92)	21.85 [15.83–28.78] (210)	12.62 [8.57–16.84] (88)	4.88 [2.96–8.24] (165)	<0.0001	<0.0001
Proteobacteria (%)	4.52 [2.14–7.16] (92)	8.50 [5.70–12.67] (210)	12.62 [8.57–16.84] (88)	22.28 [16.24–28.64] (165)	<0.0001	<0.0001
IL-6 (pg/mL)	1.46 [1.04–1.99] (117)	2.76 [2.00–3.86] (325)	6.34 [4.13–9.85] (118)	11.40 [8.08–15.41] (231)	<0.0001	<0.0001
TNF-α (pg/mL)	1.92 [1.45–2.80] (117)	2.97 [2.21–4.07] (325)	5.07 [3.65–6.72] (118)	6.76 [5.32–9.13] (231)	<0.0001	<0.0001
IL-8 (pg/mL)	7.12 [4.41–9.25] (117)	11.92 [9.17–15.54] (325)	21.81 [15.58–31.37] (118)	37.44 [27.01–55.44] (231)	<0.0001	<0.0001
IL-10 (pg/mL)	1.50 [1.06–2.08] (117)	1.51 [1.05–2.00] (325)	1.59 [1.12–2.38] (118)	1.85 [1.26–2.65] (231)	<0.0001	<0.0001
MDA (µmol/L)	2.03 [1.59–2.44] (117)	2.95 [2.48–3.60] (316)	3.73 [3.20–4.59] (118)	5.11 [4.19–6.00] (231)	<0.0001	<0.0001
TAC (mmol Trolox/L)	1.38 [1.26–1.55] (117)	1.16 [1.04–1.31] (316)	1.01 [0.86–1.18] (118)	0.76 [0.63–0.88] (231)	<0.0001	<0.0001
GSH/GSSG ratio	67.41 [58.52–79.85] (117)	52.03 [42.93–62.50] (316)	38.73 [28.96–47.18] (118)	24.75 [18.49–31.03] (231)	<0.0001	<0.0001
SOD (U/mL)	7.86 [6.68–9.44] (117)	6.62 [5.73–7.95] (316)	5.34 [4.34–6.33] (118)	4.28 [3.65–5.34] (231)	<0.0001	<0.0001
Catalase (kU/L)	52.75 [44.02–62.76] (117)	46.61 [38.94–54.54] (316)	37.49 [30.18–46.55] (118)	27.78 [20.83–36.91] (231)	<0.0001	<0.0001
*Streptococcus* *thermophilus* detected (%)	59.0% (69/117)	43.2% (134/310)	16.9% (20/118)	6.5% (15/231)	<0.0001	<0.0001
*Enterococcus* *faecium* detected (%)	52.1% (61/117)	31.6% (98/310)	19.5% (23/118)	9.5% (22/231)	<0.0001	<0.0001

**Table 2:** Proposed composite triad categories (hypothesis-generating framework).

Category	UA Status	6-SMT Status	Dysbiosis	Interpretation
A (triad-positive, high-risk)	Undetectable/very low	Critical reduction	Severe/selective dysbiosis	Amplified disruption pattern
B (intermediate)	Reduced but detectable	Moderate reduction	Moderate dysbiosis	Intermediate disruption pattern
C (triad-negative)	Normal range	Normal levels	Mild or no evidence of marked dysbiosis	Relatively preserved axis

**Table 3:** Cohort descriptions and clinical definitions (summary).

Cohort	*n*	Age (Years)	Clinical Definition	Notes
Advanced cancer, Cancer Group 1	231	57–85	Stage IV higher-aggressiveness tumor entities	UA undetectable; 6-SMT critically reduced
Advanced cancer, Cancer Group 2	118	57–85	Stage IV lower- aggressiveness tumor entities	UA reduced; 6-SMT moderately reduced
Healthy older controls	117	57–85	No known malignancy or hypertension	Reference group; no current antihypertensive treatment
Salt-sensitive hypertension	333	57–85	Elderly salt-sensitive hypertension	Comparator group; intermediate axis disruption

## Data Availability

De-identified data supporting the findings of this study are available from the corresponding author upon reasonable request and subject to institutional and ethical approvals.

## Supplementary Materials

The following supporting information can be downloaded at: https://www.mdpi.com/article/10.3390/ijms27135753/s1.

## References

[1] Garcia-Villalba R., Gimenez-Bastida J.A., Cortes-Martin A., Avila-Galvez M.A., Tomas-Barberan F.A., Selma M.V., Espin J.C., Gonzalez-Sarrias A. (2022). Urolithins: A Comprehensive Update on Their Metabolism, Bioactivity, and Associated Gut Microbiota. Mol. Nutr. Food Res..

[2] Selma M.V., Gonzalez-Sarrias A., Salas-Salvado J., Andres-Lacueva C., Alasalvar C., Orem A., Tomas-Barberan F.A., Espin J.C. (2018). The Gut Microbiota Metabolism of Pomegranate or Walnut Ellagitannins Yields Two Urolithin-Metabotypes That Correlate with Cardiometabolic Risk Biomarkers: Comparison between Normoweight, Overweight-Obesity and Metabolic Syndrome. Clin. Nutr..

[3] Iglesias-Aguirre C.E., Garcia-Villalba R., Beltran D., Frutos-Lison M.D., Espin J.C., Tomas-Barberan F.A., Selma M.V. (2023). Gut Bacteria Involved in Ellagic Acid Metabolism To Yield Human Urolithin Metabotypes Revealed. J. Agric. Food Chem..

[4] Piwowarski J.P., Kiss A.K., Granica S., Moeslinger T. (2015). Urolithins, Gut Microbiota-Derived Metabolites of Ellagitannins, Inhibit LPS-Induced Inflammation in RAW 264.7 Murine Macrophages. Mol. Nutr. Food Res..

[5] Gimenez-Bastida J.A., Gonzalez-Sarrias A., Larrosa M., Tomas-Barberan F.A., Espin J.C., Garcia-Conesa M.T. (2012). Ellagitannin Metabolites, Urolithin A Glucuronide and Its Aglycone Urolithin A, Ameliorate TNF-alpha-Induced Inflammation and Associated Molecular Markers in Human Aortic Endothelial Cells. Mol. Nutr. Food Res..

[6] Chen P., Guo Z., Chen F., Wu Y., Zhou B. (2022). Recent Advances and Perspectives on the Health Benefits of Urolithin B, A Bioactive Natural Product Derived from Ellagitannins. Front. Pharmacol..

[7] Garay-Mayol B., Gimenez-Bastida J.A., Lopez-Canovas D.J., Poveda-Lora S., Navarro-Orcajada S., Brito M.A., Nunes dos Santos C., Espin J.C., Avila-Galvez M.A., Gonzalez-Sarrias A. (2025). Urolithins and Their Phase II Conjugates Cross the Blood-Brain Barrier and Exert a Stimulus-Dependent Anti-Inflammatory Effect on Microglial Cells by Inhibiting NF-kB Nuclear Translocation. Food Funct..

[8] An L., Lu Q., Wang K., Wang Y. (2023). Urolithins: A Prospective Alternative against Brain Aging. Nutrients.

[9] Hou Y., Chu X., Park J.H., Zhu Q., Hussain M., Li Z., Madsen H.B., Yang B., Wei Y., Wang Y. (2024). Urolithin A Improves Alzheimer’s Disease Cognition and Restores Mitophagy and Lysosomal Functions. Alzheimer’s Dement..

[10] Fan X., Jin Y., Chen G., Ma X., Zhang L. (2021). Gut Microbiota Dysbiosis Drives the Development of Colorectal Cancer. Digestion.

[11] Niekamp P., Kim C.H. (2023). Microbial Metabolite Dysbiosis and Colorectal Cancer. Gut Liver.

[12] Wei Y.-F., Huang M.-S., Huang C.-H., Yeh Y.-T., Hung C.-H. (2022). Impact of Gut Dysbiosis on the Risk of Non-Small-Cell Lung Cancer. Int. J. Environ. Res. Public Health.

[13] Tarique M., Ali A.H., Kizhakkayil J., Liu S.-Q., Oz F., Dertli E., Kamal-Eldin A., Ayyash M. (2024). Exopolysaccharides from Enterococcus faecium and Streptococcus thermophilus: Bioactivities, Gut Microbiome Effects, and Fermented Milk Rheology. Food Chem. X.

[14] Huang Y.-Y., Lu Y.-H., Liu X.-T., Wu W.-T., Li W.-Q., Lai S.-Q., Aadil R.M., Rajoka M.S.R., Wang L.-H., Zeng X.-A. (2024). Metabolic Properties, Functional Characteristics, and Practical Application of Streptococcus thermophilus. Food Rev. Int..

[15] Im E.J., Lee H.H.-Y., Kim M., Kim M.-K. (2023). Evaluation of Enterococcal Probiotic Usage and Review of Potential Health Benefits, Safety, and Risk of Antibiotic-Resistant Strain Emergence. Antibiotics.

[16] Schernhammer E.S., Berrino F., Krogh V., Secreto G., Micheli A., Venturelli E., Sieri S., Sempos C.T., Cavalleri A., Schunemann H.J. (2008). Urinary 6-Sulfatoxymelatonin Levels and Risk of Breast Cancer in Postmenopausal Women. J. Natl. Cancer Inst..

[17] Brown S.B., Hankinson S.E., Eliassen A.H., Reeves K.W., Qian J., Arcaro K.F., Wegrzyn L.R., Willett W.C., Schernhammer E.S. (2015). Urinary Melatonin Concentration and the Risk of Breast Cancer in Nurses’ Health Study II. Am. J. Epidemiol..

[18] Wu A.H., Wang R., Koh W.-P., Stanczyk F.Z., Lee H.-P., Yu M.C. (2008). Sleep Duration, Melatonin and Breast Cancer among Chinese Women in Singapore. Carcinogenesis.

[19] Mirick D.K., Bhatti P., Chen C., Nordt F., Stanczyk F.Z., Davis S. (2013). Night Shift Work and Levels of 6-Sulfatoxymelatonin and Cortisol in Men. Cancer Epidemiol. Biomark. Prev..

[20] Bartsch C., Bartsch H., Schmidt A., Ilg S., Bichler K.H., Fluchter S.H. (1992). Melatonin and 6-Sulfatoxymelatonin Circadian Rhythms in Serum and Urine of Primary Prostate Cancer Patients: Evidence for Reduced Pineal Activity and Relevance of Urinary Determinations. Clin. Chim. Acta.

[21] Tai S.-Y., Huang S.-P., Bao B.-Y., Wu M.-T. (2016). Urinary Melatonin-Sulfate/Cortisol Ratio and the Presence of Prostate Cancer: A Case-Control Study. Sci. Rep..

[22] Vaselkiv J.B., Cheng I., Chowdhury-Paulino I.M., Gonzalez-Feliciano A.G., Wilkens L.R., Hauksdottir A.M., Eiriksdottir G., Le Marchand L., Haiman C.A., Valdimarsdottir U. (2022). Urinary 6-Sulfatoxymelatonin Levels and Prostate Cancer Risk among Men in the Multiethnic Cohort. Cancer Epidemiol. Biomark. Prev..

[23] Chiolero A., Maillard M., Nussberger J., Brunner H.R., Burnier M. (2000). Proximal Sodium Reabsorption: An Independent Determinant of Blood Pressure Response to Salt. Hypertension.

[24] Schoner W., Scheiner-Bobis G. (2007). Endogenous and Exogenous Cardiac Glycosides: Their Roles in Hypertension, Salt Metabolism, and Cell Growth. Am. J. Physiol. Cell Physiol..

[25] Anderson D.E., Fedorova O.V., Morrell C.H., Longo D.L., Kashkin V.A., Metzler J.D., Bagrov A.Y., Lakatta E.G. (2008). Endogenous Sodium Pump Inhibitors and Age-Associated Increases in Salt Sensitivity of Blood Pressure in Normotensives. Am. J. Physiol. Regul. Integr. Comp. Physiol..

[26] Sumner L.W., Amberg A., Barrett D., Beale M.H., Beger R., Daykin C.A., Fan T.W.-M., Fiehn O., Goodacre R., Griffin J.L. (2007). Proposed Minimum Reporting Standards for Chemical Analysis: Chemical Analysis Working Group (CAWG) Metabolomics Standards Initiative (MSI). Metabolomics.

[27] Katajamaa M., Oresic M. (2007). Data Processing for Mass Spectrometry-Based Metabolomics. J. Chromatogr. A.

