# Supplementary material for: A Dysbiosis–Urolithin A Depletion–Low 6-Sulfatoxymelatonin Triad as a Composite Biomarker Framework Across Age-Related Vascular and Malignant Disease States
Source: Int J Mol Sci. 2026 Jun 25;27(13):5753. doi: 10.3390/ijms27135753 (PMC13361097; doi:10.3390/ijms27135753)
Supplement: Supplementary file 1 [file ijms-27-05753-s001.zip › ijms-4362479-supplementary.pdf]

Supplementary Materials

Manuscript ID: ijms-4362479

A Dysbiosis-Urolithin A Depletion-Low 6-Sulfatoxymelatonin Triad as a Composite Biomarker Framework across Age-Related Vascular and Malignant Disease States

This supplementary file provides methods-oriented clarification requested during peer review. It is intentionally conservative: where quantitative assay-performance or patient-level covariate data were not uniformly available in the current revision records, the limitation is stated rather than replaced with unverifiable values.

Supplementary Method S1. Targeted quantification of urolithin A and lower-reporting-limit handling

Plasma and stool UA were measured as targeted analytes using HPLC with an authentic reference standard. Plasma was analyzed as total UA after enzymatic hydrolysis to capture conjugated forms. In the analytic summary, plasma UA values of 0.05 nmol/L and stool UA values of 0.50 nmol/g represent lower reporting limits. These observations are treated as left-censored lower-reporting-limit values and are not interpreted as true zero. For descriptive tables and nonparametric group comparisons, lower-reporting-limit values were retained at the recorded reporting limit.

Formal calibration-curve parameters, recovery, intra-/inter-assay coefficients of variation, and complete LOD/LOQ documentation were not uniformly available in the current revision files. This is reported as a limitation in the main manuscript. The authors should add full assay validation tables if those laboratory records are available before final submission.

Supplementary Method S2. 24 h urinary 6-sulfatoxymelatonin

Urinary 6-SMT was assessed from 24 h urine collections and expressed as total daily excretion (ng/day). Creatinine plausibility checks were used to support collection completeness. Because 6-SMT is influenced by renal function, sleep-wake timing, nighttime light exposure, psychological stress, and medications including beta-blockers, the revised manuscript interprets 6-SMT as a surrogate biomarker readout rather than direct evidence of circadian disruption.

Supplementary Method S3. Microbiome assessment and dysbiosis score

Dysbiosis assessment combined targeted culture-based indicators with sequencing-based profiling where available. The revised manuscript distinguishes these methods clearly. The 0-10 dysbiosis score is an investigator-defined exploratory composite index, not a validated microbiome biomarker. Higher scores indicate a more disrupted profile.

Supplementary Table S1. Exploratory dysbiosis score components.

Component	Dysbiotic direction	Score contribution
Urolithin-producing taxa	Absent or markedly reduced	0 or 2 points
Streptococcus thermophilus	Absent/not detected	0 or 2 points
Enterococcus faecium	Absent/not detected	0 or 2 points
Bacteroidetes relative abundance	Depletion relative to healthy controls	0, 1, or 2 points
Proteobacteria relative abundance	Enrichment relative to healthy controls	0, 1, or 2 points

When sequencing-derived phylum-level values were unavailable, the culture-only score was calculated and rescaled to 0-10. This rule improves reporting transparency but does not validate the score. External validation, batch-effect assessment, and reproducibility testing remain necessary.

Supplementary Method S4. Statistical notes

The ordered analytic sequence was healthy older controls -> salt-sensitive hypertension -> Cancer Group 2 -> Cancer Group 1. Continuous variables were summarized as median [IQR] and compared using Kruskal-Wallis tests; categorical variables were compared using chi-square or Fisher's exact tests. Ordered-trend p-values were based on rank-based trend testing for continuous variables and linear-by-linear association testing for categorical variables. Spearman methods were used for correlations because biomarker distributions were non-normal and UA included lower-reporting-limit values. Benjamini-Hochberg false-discovery-rate control was specified for exploratory endpoint families.

The following variables were not fully harmonized across source records and therefore are not used for definitive adjusted modeling in this revision: diet and ellagitannin intake, BMI, sleep quality, chronotype, renal function, hepatic function,

antibiotics, probiotics, chemotherapy, corticosteroids, beta-blockers, treatment line, performance status, cachexia status, and nighttime light exposure.

Supplementary Method S5. Operational triad thresholds

The operational thresholds below are data-derived and exploratory. They are intended to make the descriptive framework reproducible, not to establish clinical cutoffs.

Supplementary Table S2. Exploratory operational thresholds.

Domain	Relatively preserved	Intermediate	Highly disrupted
Plasma UA	>=1.38 nmol/L	>0.05 to <1.38 nmol/L	<=0.05 nmol/L lower reporting limit
Stool UA	>=10.14 nmol/g	>0.50 to <10.14 nmol/g	<=0.50 nmol/g lower reporting limit
24 h urinary 6-SMT	>=18.89 ng/day	5 to <18.89 ng/day	<5 ng/day
Dysbiosis score	<3.0	3.0 to <6.0	>=6.0

Supplementary Table S3. STROBE-oriented reporting checklist

STROBE item	Reporting element	Location in revised manuscript
1a-1b	Study design and balanced summary	Title page; Abstract
2-3	Background, rationale, objectives	Introduction
4-5	Study design and setting	Materials and Methods 4.1-4.3
6-8	Participants, variables, data sources	Materials and Methods 4.1-4.7
9	Bias and confounding considerations	Discussion; Materials and Methods 4.8
10-12	Study size, quantitative variables, statistical methods	Results; Materials and Methods 4.8
13-17	Participants, descriptive data, outcomes, main results	Results; Tables 1A-B
18-21	Interpretation, limitations, generalizability	Discussion; Conclusions
22	Funding and disclosures	Funding; Conflicts of Interest
